# Successful Vaginal Delivery after External Cephalic Version in a Woman with a Large Partial Uterine Septum

**DOI:** 10.1155/2021/9912271

**Published:** 2021-05-19

**Authors:** Kristen E. Park, Nicole L. Vestal, Michael S. Awadalla, Sharon A. Winer

**Affiliations:** LAC+USC Medical Center, 2020 Zonal Avenue, IRD Room 533, Los Angeles, CA 90033, USA

## Abstract

Septate uteri have been associated with adverse pregnancy outcomes including spontaneous abortion, preterm delivery, and malpresentation. It is unclear if uterine septa are associated with infertility. Although some studies have shown improved pregnancy outcomes after septum resection, indications for resection are not well established. We describe a case of a woman with a large partial uterine septum diagnosed during workup for infertility who conceived without septum resection. Both of her subsequent pregnancies were initially breech presentations for which the patient underwent external cephalic version followed by full-term vaginal deliveries. This case adds evidence that an unresected uterine septum should not be considered a contraindication to external cephalic version.

## 1. Introduction

Congenital uterine anomalies are common in the general population, with an estimated incidence ranging from 1 to 15 per 1000 women [1]. They occur due to disruptions of Mullerian duct development between the 6^th^ and 11^th^ weeks of gestation [2, 3]. A septate uterus is a uterus divided by a longitudinal band of tissue that may extend from the uterine fundus partially or completely to the cervix. There is no universally accepted definition of a uterine septum. The American Society for Reproductive Medicine defines a septum as an indentation greater than 1.5 cm depth that forms an angle less than 90 degrees [4]. The classification system presented by the European Society of Human Reproduction and Embryology and the European Society for Gynecological Endoscopy defines a septum as an internal indentation greater than 50% of the uterine wall thickness [5]. Diagnosing a uterine septum requires assessment of the anatomy of the uterine cavity and external uterine contour to distinguish a uterine septum from a bicornuate uterus. The uterine cavity can be assessed with transvaginal ultrasound, hydrosonogram, hysterosalpingogram, hysteroscopy, and/or MRI. The external uterine contour can be assessed with 2D or 3D transvaginal ultrasound, MRI, or laparoscopy [2, 4].

A septate uterus is clinically significant because it has been shown to be associated with adverse pregnancy outcomes including spontaneous abortion, preterm delivery, and malpresentation [4, 6]. Although there is inconclusive evidence on the association of septate uteri and infertility, studies have shown that septum removal can improve pregnancy rates [4, 7–9].

There is limited data to guide management of breech presentation in women with a uterine septum. A recent Practice Bulletin by the American College of Obstetricians and Gynecologists (ACOG) on external cephalic versions (ECVs) does not discuss considerations related to women with uterine septa [10]. Varying rates of ECV success have been reported in the general obstetric population. A meta-analysis of 85 studies reported a pooled success rate of 58% [11–14]. Although ECVs play a significant role in reducing cesarean delivery rates, there is limited data on ECV success rates in women with uterine abnormalities.

This case is an example of a woman with a large partial uterine septum and primary infertility who conceived naturally and had two full-term vaginal deliveries. Both vaginal deliveries were achieved after ECVs performed for breech presentation.

## 2. Case Presentation

The patient initially presented as an 18-year-old nulligravida with a chief complaint of 2 years of infertility and a known uterine septum. Gynecological history was significant for menarche at the age of 13, regular monthly menses, and a history of treated chlamydia. She had a history of congenital pulmonary stenosis treated with 2 angioplasties and hypertension managed with losartan. Her most recent echocardiogram showed an ejection fraction of 60-65% and no significant valvular disease.

**Figure 1:**
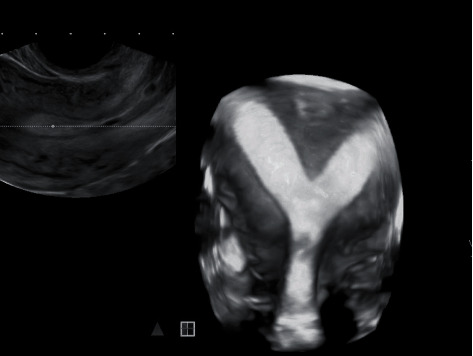
3D transvaginal ultrasound prior to conception demonstrates a large uterine septum. There is no fundal indentation seen.

**Figure 2:**
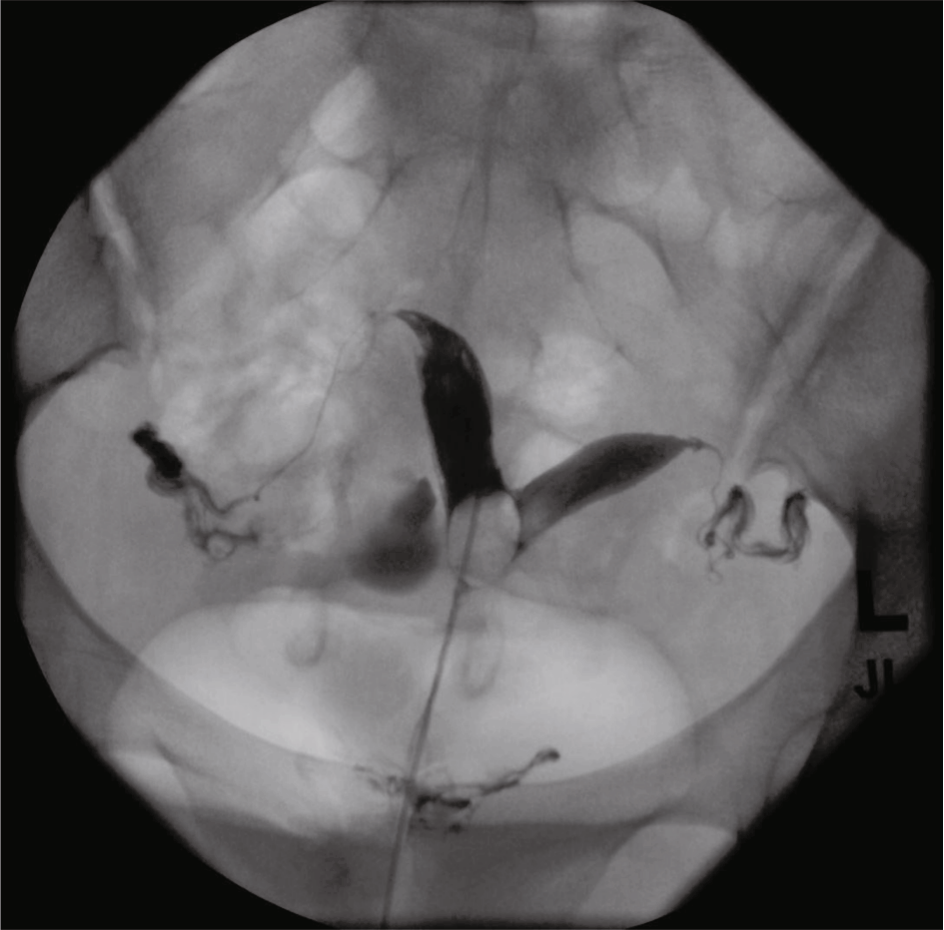
Hysterosalpingogram demonstrating two uterine cavities with patent fallopian tubes. It is not possible to differentiate between a septate uterus and a bicornuate uterus from this image because the presence or absence of a fundal indentation cannot be observed.

On examination, she was 165 cm tall and weighed 58 kg with a BMI of 21 kg/m^2^. Her vital signs were normal. A pelvic exam showed an anteverted, 8-week size uterus. Transvaginal ultrasound revealed a uterus measuring 7.1 cm length × 8.1 cm width × 4.6 cm depth with normal appearing ovaries. On 3D transvaginal ultrasound, the uterine septum had a 2 cm deep indentation with an angle of 65° (Figure 1). A hysterosalpingogram showed two uterine cornua with normal appearing and patent fallopian tubes (Figure 2). Laboratory studies were unremarkable and included an estradiol level of 81 pg/mL, a follicle stimulating hormone level of 2.8 mIU/mL, a luteinizing hormone level of 11.1 mIU/mL, and a thyroid stimulating hormone level of 0.860 *μ*IU/mL.

**Figure 3:**
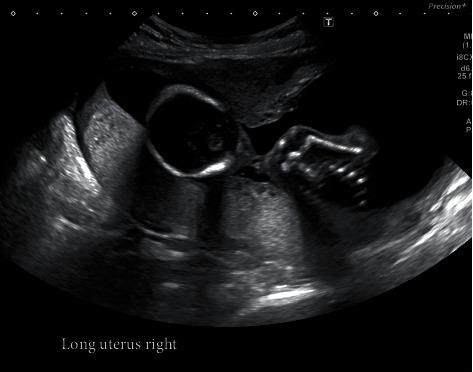
This transabdominal ultrasound image shows a right parasagittal plane at 18 weeks of gestation during the first pregnancy. A posterior placenta can be seen. Part of the uterus which appears to be a portion of the septum is seen anteriorly abutting the fetal head.

She conceived her first pregnancy spontaneously 2 months after the hysterosalpingogram was performed. Part of the uterine septum can be seen on an 18-week obstetrical ultrasound performed in this first pregnancy (Figure 3). During her first pregnancy, she was diagnosed with preeclampsia with severe features at 37 weeks of gestation at an outside hospital. An ECV was performed, followed by induction of labor and an uncomplicated vaginal delivery.

The patient then conceived her second pregnancy spontaneously 4 months after her first delivery. She received prenatal care by an outside clinic that does not offer ECVs and was referred to our obstetrical triage unit for the procedure. On presentation to our hospital, she was a 20-year-old gravida 2 para 1001 at 37 weeks and 6 days of gestational age with confirmed breech presentation and a posterior placenta. The estimated fetal weight by ultrasound was 3003 grams, and maximum vertical amniotic fluid pocket was 5.7 cm. Risks and benefits of an ECV were discussed with the patient, and consent was obtained. At 38 weeks and 0 days, an ECV was performed in the operating room under epidural anesthesia with administration of 0.25 mg subcutaneous terbutaline. The fetus was in frank breech position with the fetal head in the maternal right upper quadrant and back facing up and to the maternal left. The fetal rump was elevated out of the pelvis while pressure was simultaneously applied to the fetal head to direct it towards the pelvis. The ECV was successful on the second attempt, and anti-D immune globulin was administered after the procedure.

After the ECV, the patient was discharged home and scheduled to return for induction of labor at 39 weeks 0 days. When she returned for induction of labor, breech position was again observed by ultrasound. An epidural was placed, ECV was performed again in the operating room easily and without complications, and the patient was transferred to labor and delivery for induction of labor. During labor, the patient was diagnosed with preeclampsia with severe blood pressures and started on magnesium sulfate for seizure prophylaxis.

A singleton male in cephalic position was delivered vaginally at 39 weeks 2 days of gestation weighing 3510 grams with Apgar scores of 8 at 1 minute and 9 at 5 minutes. After delivery of the fetus and placenta, there were retained membranes that did not deliver with gentle traction. Manual uterine exploration revealed membranes firmly adhered in the right cornu which was felt to have contracted around the membranes holding them in place. The membranes were removed manually with traction. Manual uterine exploration afterwards revealed a thick uterine septum consistent with previous 3D TVUS and hysterosalpingogram findings. The postpartum course was uncomplicated, and another dose of anti-D immune globin was administered on postpartum day 2 since the infant was Rh positive. The mother and infant were both discharged on postpartum day 2.

## 3. Discussion

Septate uteri constitute roughly 35% of congenital uterine abnormalities, making them the most common type of Mullerian anomaly [15]. In spite of this, there is still much debate surrounding its pathophysiology, diagnosis, and treatment. In cases of breech presentation and a septate uterus, there are no specific guidelines for management.

Prior studies have found evidence that hysteroscopic septum incision may improve clinical pregnancy rates and improve outcomes with some researchers proposing that improvement may be due to uterine cavity remodeling into a normal size and shape postoperation [4, 16–18]. However, a recent randomized controlled trial found compared to expected management, septum resection did not improve live birth rates, decrease pregnancy loss, or decrease preterm birth rates. One uterine perforation occurred in a patient randomized to septum resection. This study was limited by a sample size of 40 in each group [19].

There is a lack of guidance on management of breech presentation in patients with uterine abnormalities. There is evidence that ECVs are more successful in patients of older age, higher parity, higher amniotic fluid index, posteriorly positioned placentas, and low bladder volume [13, 20]. However, studies often exclude patients with uterine abnormalities [12, 13]. Although there are several reported cases of successful and unsuccessful ECV attempts in women with uterus didelphys, there is much less information on ECV attempts in women with septa [21–23]. This case demonstrates that ECVs can be successfully completed in patients with uterine septa. Future research into ECV should consider including patients with uterine abnormalities and reporting outcomes for these cases to better understand success rates.

In this case, the fetus reverted back to breech position after the first successful ECV. One large retrospective study found that reversion after successful ECV occurred in about 2% of cases [24]. It is unknown if this rate differs in cases of uterine malformations. ACOG recommends considering a retrial of ECV in cases where the fetus reverts to breech and reports that there is no evidence to support induction of labor after an ECV prior to 39 weeks [10]. One recent study found that a second ECV after a successful first ECV with spontaneous reversion was successful in approximately 75% of cases but resulted in a higher rate of cesarean deliveries as compared to patients with a successful first ECV and no reversion. However, patients with uterine malformations were not included in this study [25].

Further research into ECV reversion rates in cases of uterine malformation will help guide management in this subset of patients. For example, if the rate of reversion is higher in cases of uterine malformations, it may be better to schedule ECV at 39 weeks (rather than 37 or 38 weeks) with a plan to induce labor if the version is successful or deliver by cesarean section if unsuccessful.

In summary, we present a case of a woman with an unresected large partial uterine septum who had two term vaginal deliveries after ECVs. Although septum resection has been shown to reduce the risk of obstetrical complications, more information is needed on how septum size and shape impacts these risks. Although more studies that include women with uterine abnormalities are needed to create guidelines, our case suggests that a large partial uterine septum should not be considered a contraindication to ECV.

## Data Availability

No data were used to support this study.

## References

[1] Valle R. F., Ekpo G. E. (2013). Hysteroscopic metroplasty for the septate uterus: review and meta-analysis. *Journal of Minimally Invasive Gynecology*.

[2] Behr S. C., Courtier J. L., Qayyum A. (2012). Imaging of Müllerian duct anomalies. *Radiographics*.

[3] Troiano R. N., McCarthy S. M. (2004). Müllerian duct anomalies: imaging and clinical issues. *Radiology*.

[4] Pfeifer S., Butts S., Dumesic D. (2016). Uterine septum: a guideline. *Fertility and Sterility*.

[5] Grimbizis G. F., Campo R. (2012). Clinical approach for the classification of congenital uterine malformations. *Gynecological Surgery*.

[6] Woelfer B., Salim R., Banerjee S., Elson J., Regan L., Jurkovic D. (2001). Reproductive outcomes in women with congenital uterine anomalies detected by three-dimensional ultrasound screening. *Obstetrics & Gynecology*.

[7] Saygili-Yilmaz E., Yildiz S., Erman-Akar M., Akyuz G., Yilmaz Z. (2003). Reproductive outcome of septate uterus after hysteroscopic metroplasty. *Archives of Gynecology and Obstetrics*.

[8] Tomaževič T., Ban-Frangež H., Virant-Klun I., Verdenik I., Požlep B., Vrtačnik-Bokal E. (2010). Septate, subseptate and arcuate uterus decrease pregnancy and live birth rates in IVF/ICSI. *Reproductive Biomedicine Online*.

[9] Mollo A., de Franciscis P., Colacurci N. (2009). Hysteroscopic resection of the septum improves the pregnancy rate of women with unexplained infertility: a prospective controlled trial. *Fertility and Sterility*.

[10] Baxi L. (2020). External cephalic version: ACOG Practice Bulletin number 221. *Obstetrics and Gynecology*.

[11] Homafar M., Gerard J., Turrentine M. (2020). Vaginal delivery after external cephalic version in patients with a previous cesarean delivery: a systematic review and meta-analysis. *Obstetrics & Gynecology*.

[12] Impey O. R., Greenwood C. E., Impey L. W. (2018). External cephalic version after previous cesarean section: a cohort study of 100 consecutive attempts. *European Journal of Obstetrics & Gynecology and Reproductive Biology*.

[13] Levin G., Rottenstreich A., Weill Y., Pollack R. N. (2019). External cephalic version at term: a 6-year single-operator experience. *Birth*.

[14] Grootscholten K., Kok M., Oei S. G., Mol B. W., van der Post J. A. (2008). External cephalic version–related risks: a meta-analysis. *Obstetrics & Gynecology*.

[15] Grimbizis G. F., Camus M., Tarlatzis B. C., Bontis J. N., Devroey P. (2001). Clinical implications of uterine malformations and hysteroscopic treatment results. *Human Reproduction Update*.

[16] Grimbizis G. F. (2019). The pathophysiology of septate uterus. *BJOG: An International Journal of Obstetrics & Gynaecology*.

[17] Chan Y. Y., Jayaprakasan K., Tan A., Thornton J. G., Coomarasamy A., Raine-Fenning N. J. (2011). Reproductive outcomes in women with congenital uterine anomalies: a systematic review. *Ultrasound in Obstetrics and Gynecology*.

[18] Detti L. (2014). Ultrasound assessment of uterine cavity remodeling after surgical correction of subseptations. *American Journal of Obstetrics and Gynecology*.

[19] Rikken J. F. W., Kowalik C. R., Emanuel M. H. (2021). Septum resection versus expectant management in women with a septate uterus: an international multicentre open-label randomized controlled trial. *Human Reproduction*.

[20] Levin G., Rottenstreich A., Weill Y., Pollack R. N. (2018). The role of bladder volume in the success of external cephalic version. *European Journal of Obstetrics & Gynecology and Reproductive Biology*.

[21] Ratzersdorfer J., Abdelhak Y. (2017). Vaginal birth after two previous caesarean deliveries in a patient with uterus didelphys and an interuterine septal defect. *Case Reports*.

[22] Ross C., El-Hassan H., Lakasing L. (2018). Uterus didelphys: two pregnancies, two term breech caesarean deliveries. *Case Reports*.

[23] Mirzai S., Wolf S. B., Mili S., Rifai A. O. (2019). Successful external cephalic version in a patient with uterus didelphys and fetal malpresentation. *BMJ Case Reports*.

[24] Melo P., Georgiou E. X., Hedditch A., Ellaway P., Impey L. (2019). External cephalic version at term: a cohort study of 18 years’ experience. *BJOG: An International Journal of Obstetrics & Gynaecology*.

[25] Reicher L., Lavie A., Fouks Y. (2021). Efficacy of a second external cephalic version (ECV) after a successful first external cephalic version with subsequent spontaneous reinversion to breech presentation: a retrospective cohort study. *Archives of Gynecology and Obstetrics*.

